# Development and Validation of Social Trust Scale for Chinese Adolescents (STS-CA)

**DOI:** 10.3390/bs15111436

**Published:** 2025-10-22

**Authors:** Youling Bai, Luoxuan Li, Yuhan Yang, Yanling Liu

**Affiliations:** 1 Faculty of Psychology, Southwest University, Chongqing 400715, China; byling819@email.swu.edu.cn (Y.B.); 17830052776@163.com (L.L.); yyh1128@email.swu.edu.cn (Y.Y.); 2 Mental Health Education Research Center, Southwest University, Chongqing 400715, China

**Keywords:** social trust, adolescents, validity, reliability, scale development

## Abstract

Social trust is a crucial factor influencing adolescents’ mental health and serves as a cornerstone for social stability. However, there is a lack of a reliable measurement tool specifically designed to assess the psychological characteristics of adolescents’ social trust. This study aimed to develop and validate the Chinese Adolescent Social Trust Scale (STS-CA). Semi-structured interviews were conducted with 45 adolescents (aged 12 to17 years) to generate an initial pool of scale items. Subsequently, eighteen psychological experts evaluated the content validity, and the scale was revised based on their feedback, resulting in a preliminary version. A total of 2036 secondary school students were randomly divided into Sample 1 and Sample 2. Sample 1 (*N*1 = 1018) was used in item analysis and exploratory factor analysis (EFA). Sample 2 (*N*2 = 1018) was utilized for confirmatory factor analysis (CFA). Sample 3 (*N*3 = 1214) was recruited to assess the scale’s reliability, validity, and measurement equivalence. Two months later, test–retest reliability analysis was assessed using Sample 4 (*N*4 = 303). The final STS-CA consists of 27 items covering four factors, namely trust in relatives, trust in friends, trust in strangers, and trust in organizations. The scale demonstrated good internal consistency reliability, test–retest reliability, convergent validity, and discriminant validity. Correlations between the STS-CA subscale scores and external criterion variables—interpersonal trust, trust propensity, and life satisfaction—supported criterion validity. Additionally, the scale exhibited good measurement equivalence across gender and educational stages. Overall, our findings demonstrate that the STS-CA is a reliable and valid instrument for assessing social trust levels among Chinese adolescents.

## 1. Introduction

Trust plays a critical role in individual development and the orderly functioning of society (Thielmann & Hilbig, 2015). With the rapid transformation and development of Chinese society, the issue of social trust has become increasingly prominent (Gong, 2015), drawing the attention of many scholars. Social trust refers to an individual’s positive psychological expectation or perceived credibility regarding the reliability of both others and broader societal systems, such as institutions and organizations (Jensen & Svendsen, 2011). Adolescence is a crucial developmental stage for the formation of social trust (Wray-Lake & Flanagan, 2012). The level of social trust during this period not only significantly influences adolescents’ mental health (Xiong et al., 2023; Zhao et al., 2024) but is also closely linked to broader social stability and development (Fink & Kessler, 2010).

Empirical research has shown that adolescent social trust is significantly associated with self-esteem (Liu et al., 2018) and psychosocial adjustment (Sutter & Kocher, 2007). Longitudinal research conducted in Western contexts has found that early adolescents report higher levels of social trust than their middle and late adolescent counterparts, demonstrating a noticeable decline even within one year (Flanagan & Stout, 2010). Furthermore, a longitudinal study from Sweden involving five distinct youth cohorts (aged 13 to 28) indicated that social trust was particularly unstable between the ages of 13 and 15 and tended to stabilize with age (Abdelzadeh & Lundberg, 2017). However, the developmental characteristics of social trust among Chinese adolescents remain insufficiently explored. Existing studies within the Chinese context have predominantly focused on interpersonal trust (e.g., Gong, 2015; Li et al., 2025), with limited attention given to broader dimensions of social trust. Consequently, accurate and reliable psychological measurement tools are a basic prerequisite for promoting the study of social trust levels and characteristics among Chinese adolescents.

### 1.1. Overview of Social Trust Structure

Although social trust has been extensively studied across various disciplines, a consensus on its structural definition remains elusive. Early and representative classifications include generalized trust versus particularized trust (Rotter, 1971), interpersonal trust versus organizational trust (Bai, 2006; Paxton, 1999), and interpersonal trust versus institutional trust (Luhmann, 2005; Putnam, 2001). Specifically, Rotter (1971) distinguished between generalized trust and particularized trust based on the criteria for determining social trust. Generalized trust refers to trust relationships established through the guarantee of credit contracts and legal norms, where establishing trust relationships requires the trust subject’s strict adherence to the credit contract. Particularized trust, in contrast, refers to relationships founded on special kinship ties (e.g., blood, familial, or friendship bonds) and guaranteed by informal institutional arrangements such as morality or ideology. Luhmann (2005) categorized social trust into interpersonal trust and system trust from a sociological perspective, arguing that the former is based on familiarity and emotional ties between individuals; while the latter is built upon social norms and systems, reflecting trust in groups, institutional organizations, and societal structures. This dichotomy has been widely adopted by academia. Paxton (1999) classified social trust into trust in people and trust in institutions from the perspective of social capital components. Based on the sources of trust, Putnam (2001) proposed the classic classification of interpersonal trust (trust in people) and institutional trust (trust in social systems and their functioning), a framework still followed by scholars. Uslaner (2003) later refined the concept of interpersonal trust, distinguishing trust in most people (generalized trust) from trust in closer individuals (particularized trust). Drawing on Fei’s concept of “the order of stratified closeness (chaxugeju)”, the structure of trust in Chinese society is described as ego-centered, diminishing in concentric circles as relational distance increases (Fei, 2008). This conceptualization underpins various dichotomous constructions of trust, such as “strong trust versus weak trust,” “interpersonal trust versus institutional trust,” and “particularized trust versus generalized trust.” From the perspective of intersubjective relationships, Bai (2006) divided trust into that between individuals and trust between individuals and organizations. Collectively, these classifications suggest that social trust encompasses interpersonal trust within the realm of interpersonal interaction (i.e., trust toward familiar and unfamiliar individuals) and organizational or institutional trust within the domain of social public life.

### 1.2. Overview of Social Trust Measurement

Among the self-report measures used to study social trust, three types of instruments have been used. First, researchers frequently utilized items from large-scale surveys like the China General Social Survey (CGSS), the World Values Survey (WVS), and the China Family Panel Studies (CFPS), employing questions such as, “whether people are generally trustworthy or whether it is best to be careful?” (Huhe, 2014; Wang et al., 2024; Xu et al., 2023). However, the validity of such single-item measures has been questioned, as it remains unclear how respondents interpret the questions or whether the item genuinely measures trust or reflects societal perceptions of trustworthy behavior (Delhey et al., 2011; Glaeser et al., 2000). Additionally, these surveys primarily serve sociological research purposes and are inadequate for capturing the psychological characteristics of adolescents’ social trust. Second, scales focusing specifically on trust or interpersonal trust have been employed. Examples include the Trust Scale (Rempel & Holmes, 1986), assessing trust within close relationships; scales measuring interpersonal trust propensity as a personality trait (Evans & Revelle, 2008); and Yamagishi and Yamagishi (1994) general trust scale, incorporating beliefs about others’ trustworthiness and preferences for trustful behavior. However, these scales primarily target individual-level or interpersonal trust and lack a comprehensive assessment of broader social trust dimensions. The most widely used among these is Rotter’s (1967) Interpersonal Trust Scale. This 25-item scale addresses trust in general and toward specific social entities (e.g., parents, teachers, and public officials). However, some items, particularly those related to “political participation” and “official elections,” are culturally specific and less applicable within the Chinese context. Furthermore, platforms like Questionnaire Star often flag such items as sensitive, complicating distribution and data collection. Third, some researchers have developed context-specific social trust questionnaires for different populations (Li, 2014; Lin, 2019). Nevertheless, these unpublished instruments lack established evidence of effectiveness and reliability. Therefore, it is particularly essential to develop a social trust scale suitable for Chinese adolescents.

### 1.3. The Present Study

Developing a scientifically valid social trust measurement tool is paramount for advancing research and assessing levels of social trust among Chinese adolescents. To date, however, research on social trust has suffered from a lack of a concise and rigorously validated scale. The primary purpose of the present study was therefore to develop and validate a scale assessing the level of social trust among native Chinese adolescents. Following a standard scale development procedure (DeVellis & Thorpe, 2021), we aimed to identify Chinese adolescents’ trust levels toward people and organizations within their society, examine the scale’s factor structure, and establish valid evidence. Specifically, the study involved the following steps: First, generating an initial item pool through semi-structured interviews, referring to the existing trust and interpersonal trust scales, and expert evaluation. Second, conducting item analysis and EFA on Sample 1 to uncover the latent factor structure. Third, performing CFA on an independent sample (Sample 2) to validate the structure identified in the EFA. Fourth, evaluating internal consistency, convergent validity, discriminant validity, and criterion-related validity using Sample 3. Finally, test–retest reliability was assessed using Sample 4. Collectively, these steps provide robust psychometric evidence supporting the STS-CA as a reliable and valid instrument for assessing Chinese adolescents’ social trust.

## 2. Methods

### 2.1. Participants

Prior to data collection, the required sample size was estimated based on established criteria: typically 5–10 or 10–20 times the number of items (Wu, 2020); test–retest samples generally require 20–30 participants (Jiang et al., 2010). The sample size required for EFA in this study should be at least 185 to 370 or 370 to 740 individuals, and the sample size required for validation factor analysis should be at least 135 to 270 or 270 to 540 individuals.

Using convenience sampling, 2100 adolescents were recruited from five middle schools in Sichuan, Chongqing, Xinjiang, Shandong, and Inner Mongolia provinces for online and offline surveys. Out of the 2047 responses retrieved, invalid questionnaires (completion time < 2 s/item or > 70% identical responses) were excluded, yielding 2036 valid responses. These were randomly split into two samples: Sample 1 (*N*1 = 1018, *M*_age_ = 14.81, *SD* = 1.64) was used for item analysis and exploratory factor analyses. The sample included 522 boys (51.3%) and 496 girls (48.7%). The participants were aged from 12 to 18 years. There were 148 (14.5%) seventh graders, 228 (22.4%) eighth graders, 117 (11.5%) ninth graders, 266 (26.1%) tenth graders, 170 (16.7%) eleventh graders, and 89 (8.7%) twelfth graders; 431 (42.3%) were only children, and 587 (57.7%) were not. In total, 378 participants were from rural areas, 276 from towns, and 364 from cities. Sample 2 (*N*2 = 1018, *M*_age_ = 14.80, *SD* = 1.62) was used for confirmatory factor analysis. Among them, 555 (54.5%) were boys and 463 (45.5%) were girls. There were 147 (14.4%) seventh graders, 241 (23.7%) eighth graders, 107 (10.5%) ninth graders, 275 (27.0%) tenth graders, 160 (15.7%) eleventh graders, and 88 (8.6%) twelfth graders; 457 (44.9%) were only children, and 561 (55.1%) were not. A total of 412 participants were from rural areas, 296 from towns, and 310 from cities.

Sample 3 (*N*3 = 1214, *M*_age_ = 14.96, *SD* = 1.76) was used to perform internal consistency reliability analysis, discriminant validity analysis, convergent validity, and criterion-related validity analysis. Convenience sampling was used to distribute 1300 questionnaires from five middle schools in Gansu Province. 1238 questionnaires were retrieved, 24 invalid responses (e.g., consecutive regular responses, incomplete items) were excluded, and 1,214 valid data were gained. Of these, 244 (20.1%) were from Grade 7, 195 (16.1%) from Grade 8, 192 (15.8%) from Grade 9, 200 (16.5%) from Grade 10, 196 (16.1%) from Grade 11, and 187 (15.4%) from Grade 12; 634 (52.2%) were boys and 580 (47.8%) were girls; 89 (7.3%) were only children, and 1125 (92.7%) were not. There were 330 participants from rural areas, 633 from towns, and 251 from cities. In terms of fathers’ education level, 156 had only completed elementary school, 514 had completed junior high school, 293 had completed high school or vocational school, 107 had gone to college, and 144 had earned a bachelor’s degree or more. For Mothers’ educational level included elementary school or less (338), junior high school (435), high school or vocational school (241), college (91) and bachelor’s degree (100).

Sample 4 (*N*4 = 303, *M*_age_ = 15.17, *SD* = 1.74, 132 girls) was used for test–retest reliability analysis. Two months later, 303 participants from Sample 3 were retested. Among them, 48 (15.8%) were from Grade 7, 44 (14.5%) from Grade 8, 52 (17.2%) from Grade 9, 51 (16.8%) from Grade 10, 55 (18.2%) from Grade 11, and 53 (17.5%) from Grade 12.

Informed consent was obtained from the school, teacher, and students for all survey procedures of the study. This study was reviewed and approved by the Research Project Ethical Review Committee of the first author’s University.

### 2.2. Scale Development

#### 2.2.1. Item Generation

The social trust scale items were generated through three methods. First, by adapting adolescents’ views or opinions about social trust from the interview data, and revising some expressions that could represent a certain dimension into questions under that dimension. Specifically, forty-five adolescents (24 boys; aged 12–17 years) were recruited through convenience sampling from two junior high schools and three senior high schools located in Chongqing, Hunan, Anhui, and the Inner Mongolia Autonomous Region. Among them, 18 were from junior high schools and 27 were from senior high schools. One-on-one semi-structured interviews were conducted with each participant, and each interview lasted approximately 20 to 30 min. The interviews centered on the following questions: ① What do you think “society” consists of when I mention the term “social trust”? From the concept of society, what do you think is social trust? What does social trust consist of? ② In your whole social life, which people (familiar people, e.g., parents, relatives, classmates, community; strangers) do you think are worth your trust? Why do you think they are trustworthy? ③ In your whole social life, which organizations (institutions, systems, media) do you feel are worthy of your trust? Why do you think they are trustworthy? Please talk about them specifically with examples. ④ In social life, do you think it is important for you to trust people and systems? Secondly, some questions were prepared based on the theoretical construction of social trust among adolescents and the definition of each dimension. Thirdly, with reference to individual items from the scale on trust and interpersonal trust (Rotter, 1967; Yamagishi & Yamagishi, 1994; Yamagishi et al., 2015). Items were refined for clarity so that they were accurate, concise, and easy to understand, while avoiding ambiguity, double meanings, and redundant expressions, resulting in a preliminary set of 94 items to measure social trust among adolescents. Subsequently, one psychology faculty member, eight doctoral students, and ten master’s students conducted a preliminary review and assessment of all the questions. The assessment included: (1) whether the items accurately reflected the content of each dimension; (2) whether the language expression was standardized and smooth, avoiding multiple meanings or ambiguities, and ensuring that it was appropriate for the adolescents’ age characteristics. Specifically, ten items with repetitions were removed, six items with similar expressions were merged, eleven items unrelated to social trust were deleted, and seven items were excluded due to ambiguity. In sum, thirty-four items were deleted at this stage, and sixty items were retained.

The scale utilized a 5-point scoring ranging from 1 (completely disagree) to 5 (completely agree). The individual responses for each item within the subscales were aggregated to yield subscale scores. These subscale scores were then cumulatively added to derive an overall scale score.

#### 2.2.2. Content Validity Assessment

To ensure the content validity of the initial scale and the relevance of the items to the theoretical conceptualization, a panel of 18 experts (11 professors, 6 associate professors, and 1 secondary school teacher) was invited to evaluate the representativeness of the sixty items and their correspondence with the dimensions of social trust using a 5-point scale (1 = “very irrelevant,” 2 = “irrelevant,” 3 =“neutral,” 4 = “relevant,” and 5 = “very relevant”). Experts were also asked to provide any modification suggestions in the designated section. Results revealed that 23 items had content validity indices (item-level content validity index, I-CVI) ranging from 0.278 to 0.722, below the threshold of 0.78 (Lyun, 1986), and thus were deleted. The remaining 37 items had I-CVI values between 0.833 and 1.00, exceeding the threshold of 0.78, and were retained. Meanwhile, the scale-level content validity index (S-CVI/Ave) of the remaining 37-item scale was 0.926, exceeding the recommended cut-off value of 0.90 (Polit & Beck, 2006), indicating excellent overall content validity. Furthermore, using convenience sampling, 50 adolescents were recruited from both junior and senior high schools in Anhui, Hunan, Guizhou, Xinjiang, Chongqing and Yunnan provinces. The participants consisted of 24 boys and 26 girls, 12 seventh-grade, 16 eighth-grade, 12 tenth-grade and 10 eleventh-grade students. All participants were asked to rate the readability and clarity of the retained thirty-seven items on a 6-point scale (1 = “completely unclear,” 6 = “completely clear”). The results showed that all items received scores ranging from 4 (somewhat clear) to 6 (completely clear).

### 2.3. Criterion-Related Validity Measures

The Interpersonal Trust Scale (ITS) was first developed by Rotter (1967) to measure levels of interpersonal trust under various situational conditions. The scale consists of 25 items scored on a 5-point Likert scale ranging from 1 (strongly disagree) to 5(strongly agree). The higher the score, the higher the level of interpersonal trust. The McDonald omega coefficient of ITS in this study was 0.718.

The Trust Scale was developed by Yamagishi and Yamagishi (1994), comprising two subscales: the Belief Subscale (General Trust) and the Preference Subscale (Trust Propensity). General trust refers to an individual’s belief in the trustworthiness of others, such as the statement, “Most people can be trusted.” Trust propensity, on the other hand, refers to an individual’s positive evaluation of others’ behavior as trustworthy, exemplified by the statement, “Even though I sometimes suffer consequences from trusting others, I still choose to trust them.” This scale consists of 9 items, rated on a 7-point Likert scale (1 = strongly disagree to 7 = strongly agree), with higher scores indicating a higher level of trust. In this study, the McDonald omega coefficient for this scale was 0.821.

The Life Satisfaction scale was developed by Diener et al. (1985). It consists of five items. Responses use a 7-point Likert scale (1  = ”completely disagree,” 7  = ”strongly agree”), with higher scale scores indicating a greater perceived level of life satisfaction. This scale has been widely used in adolescent samples (Proctor et al., 2009). In this study, the McDonald omega coefficient for this scale was 0.800.

### 2.4. Data Analyses

The data were processed using SPSS 27.0 and Mplus 8.0. Firstly, item analysis was conducted in SPSS 27.0, including critical ratio analysis and correlation analysis. Specifically, critical ratio analysis was used to rank the total social trust scores, dividing the sample into a high-score group (27% of samples with high scores) and a low-score group (27% of samples with low scores). Subsequently, independent samples *t*-tests were used to calculate the significance of the differences in the average scores of each item between the high and low groups. A correlation analysis was performed between each item and the total score of STS-CA. Secondly, an EFA, using principal axis factoring with oblimin rotation, was conducted to explore the dimensionality of STS-CA. Meanwhile, parallel analysis method (Horn, 1965) was conducted to establish the correct number of factors. Next, CFA was conducted using Mplus 8.0 to evaluate the goodness-of-fit of the model to the data, with the four factors specified as correlated. According to Hu and Bentler (1999), the following fit indices were employed: the Standardized Root Mean Squared Residual (SRMR ≤ 0.08), the Root Mean Square Error of Approximation (RMSEA; ≤ 0.06 for good, ≤ 0.08 for acceptable), the Tucker–Lewis index (TLI ≥ 0.90 for acceptable), and the Comparative Fit Index (CFI ≥ 0.90 for acceptable). Thirdly, the reliability and validity of the final scale were assessed, such as Cronbach’s alpha, McDonald’s omega, test–retest reliability, criterion-related validity, discriminant validity, and convergent validity. We assessed the scale’s convergent validity by calculating the composite reliability (CR) and a standardized factor loading. Each factor’s CR value was greater than 0.7, suggesting strong convergent validity and reliability (Hair et al., 2006). As suggested by Hair et al. (2006), standardized factor loadings were greater than 0.5, indicating an acceptable convergent validity. If the square root of average variance extracted (AVE) of a factor is greater than the absolute value of the correlation coefficient between that factor and other factors, it indicates that the scale has good discriminative validity. Finally, measurement invariance was examined to ensure the robustness and validity of the instrument using Mplus 8.0. To further examine the measurement equivalence of the STS-CA across gender and school stage, multi-group CFA was conducted according to Vandenberg and Lance’s recommended methodology (Vandenberg & Lance, 2000), including the configural invariance, metric invariance (equal factor loadings), and scalar invariance (equal intercepts), respectively. Model comparisons consisted of two main aspects: (1) the overall performance of the model fit; and (2) the assessment of measurement equivalence by comparing changes in CFI and TLI (i.e., ∆CFI and ∆TLI). When ∆CFI ≤ 0.01 and ∆TLI ≤ 0.01, RMSEA ≤ 0.015, measurement invariance should be supported (Cheung & Rensvold, 2002; Van den Berg et al., 2002).

## 3. Results

### 3.1. Common Method Bias

Before formally analyzing the data, the Harman single-factor method was used to conduct the common method bias test in the Sample 1 data. The results showed that there was a total of nine factors with eigenvalues greater than 1, and the variance variation explained by the first factor was 31.49%, which was less than the critical value of 40% (Podsakoff et al., 2003), indicating that there was no common method bias in the data of this study.

### 3.2. Item Analysis

Item analysis was conducted on Sample 1 (*N*1 = 1018), including independent samples *t*-test for both high and low scores groups on 37 items and the item-total correlation coefficient (*r*). Firstly, the independent samples *t*-test results showed that two items (ST23, ST29) failed to show statistically significant differences between the high and low scores groups (*p* > 0.05) and were therefore excluded. All other items displayed significant differences between the groups (*ps* < 0.001), and thus they were retained. Secondly, to assess the homogeneity of the scale, Pearson’s correlation test was applied to calculate the coefficient between the item scores and the total score. Correlations coefficients ranged from 0.07 to 0.72. However, the correlation coefficients for four items (ST18, ST23, ST26, and ST29) were below 0.40 (Wu, 2010), so these items were excluded. As a result, 33 items were retained (See Table 1).

### 3.3. Validity Analysis

#### 3.3.1. EFA 

After item analysis, an EFA was conducted on the 33 retained items using Sample 1. The KMO test (KMO = 0.96) and Bartlett’s test of sphericity (χ^2^ = 19030.09, *df* = 528, *p* < 0.001) indicated that the data were suitable for EFA (Pallant, 2001). EFA was conducted using principal axis factoring with oblique (promax) rotation, and items were excluded based on the following criteria: (1) commonality less than 0.35; (2) difference in absolute value of loadings on more than one factor less than 0.15. The results of the EFA indicated that item 6 had a commonality of 0.229, and items 7, 13, 34, and 35 exhibited serious cross-loadings; therefore, a second EFA was conducted after deleting items 6, 7, 13, 34, and 35, and it was found that item 30 has cross-loadings and meets the exclusion condition (2). A third EFA was conducted after removing item 30, and Bartlett’s test of sphericity showed χ^2^ = 15,411.025, *df* = 351 (*p* < 0. 001), and KMO = 0.951, indicating that the data were suitable for factor analysis. As shown in Table 2, the third factor analysis extracted four principal components, as expected. Each item had large loadings on the corresponding factor, ranging from 0.378~0.882.

**Figure 1:**
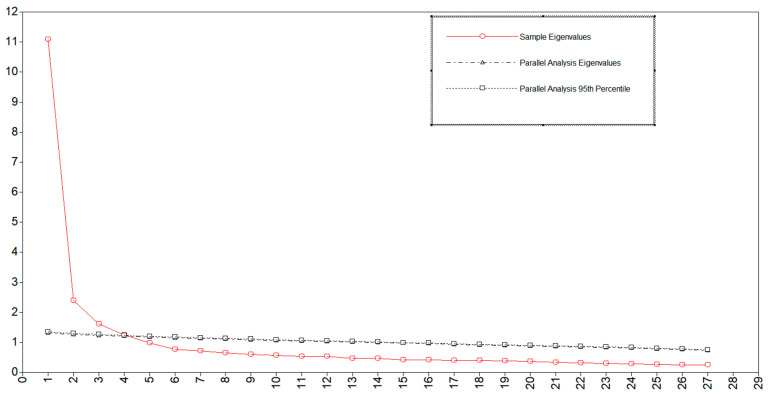
Results from parallel analysis.

In addition, the most precise method of factor retention is parallel analysis, i.e., if a factor drawn from real data explains less variation than the corresponding number of factors drawn from simulated random data, it has no value for retention and should be discarded (Ledesma & Valero-Mora, 2007). Parallel analysis of the 27 items initially identified by the EFA showed that the eigenvalues of the four factors from the real data fell on the average eigenvalue of the random data matrix, as shown in Figure 1. Therefore, four factors were retained.

In summary, combined with EFA and parallelism analysis, it was finally determined that the Social Trust scale for Chinese adolescents consists of 27 items in 4 dimensions. Among them, factor 1 mainly measures the degree of adolescents’ trust in their relatives, which is expressed as the degree to which individuals perceive the reliability, safety, and support of their parents and relatives, including items 1, 4, 16, 24 and 37, named trust in relatives. Factor 2 mainly measures adolescents’ trust in friends, which is expressed as the extent to which individuals perceive the reliability, security, and supportiveness of their friends, including items 2, 5, 14, 20, and 36, named trust in friends. Factor 3 mainly measures adolescents’ trust in strangers, which is expressed as the extent to which individuals perceive the reliability, safety, and supportiveness of strangers, including items 3, 9, 10, 21, and 22, named trust in strangers; Factor 4 mainly measures adolescents’ trust in organizations, which is expressed as the extent to which individuals perceive the fairness, usefulness, and reliability of organizational institutions and their systems, including items 8, 11, 12, 15, 17, 19, 25, 27, 28, 31, 32, and 33, named trust in organizations. Taken together, the naming of the factors is more in line with the content of the items, indicating that the scale has good structural validity.

#### 3.3.2. Confirmatory Factor Analysis (CFA)

**Figure 2:**
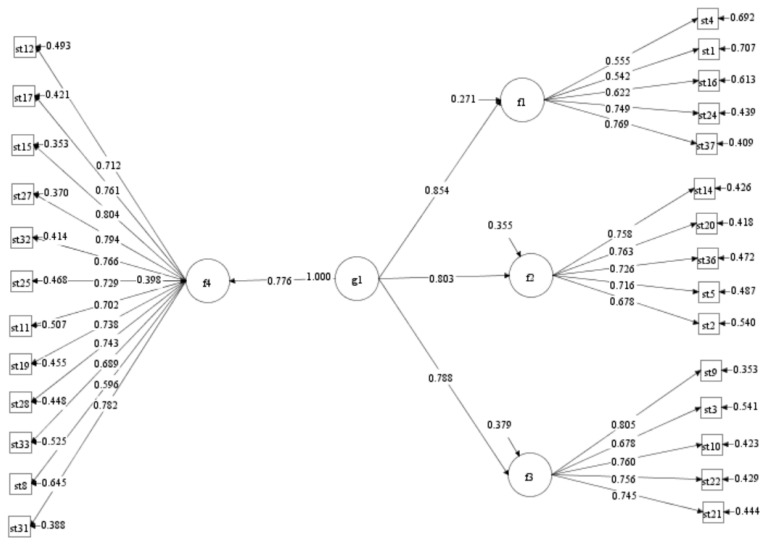
Standardized path of the four-factor model for the STS-CA (g1 = Trust in others; f1 = Trust in relatives; f2 = Trust in friends; f3 = Trust in strangers; f4 = Trust in the organization, st = social trust).

Prior to conducting the CFA, the multivariate normality (Mardia coefficient) was calculated to determine the appropriate estimation method. The results indicated significant deviations from multivariate normality (Mardia’s multivariate skewness = 12,384.31, *p* < 0.001; Mardia’s multivariate kurtosis = 117.591, *p* < 0.001), suggesting that the data deviate from multivariate normal distribution. Nevertheless, given the large sample size (*N*2 = 1018), previous research has demonstrated that the robust maximum likelihood (MLR) estimator maintains robustness against deviations from multivariate normality (Finney & DiStefano, 2006). Therefore, a CFA was conducted with Sample 2 (*N*2 = 1018) using the MLR estimator. The fit indices for the four-factor model based on the final 27 items showed a barely acceptable model fit (χ^2^/*df* = 4.124, CFI = 0.906, TLI = 0.897, RMSEA = 0.055, SRMR = 0.046) compared to the other factor models (See Table 3). To improve the goodness-of-fit of model, the residual correlation based on the modification indices were added. The results indicated that the model fitting was significantly improved (χ^2^/*df* = 3.111, CFI = 0.938, TLI = 0.931, RMSEA = 0.046, SRMR = 0.040). Therefore, the results of CFA demonstrated the validity of a four-factor model comprising 27 items. In addition, the standardized loadings of each measurement item of the four-factor model on the corresponding factors ranged from 0.542 to 0.805 (*p* < 0.001, see Figure 2), which can better explain the observed variables (Qiu, 2010). The dimensions of the scale and the corresponding items are presented in Appendix A.

#### 3.3.3. Criterion-Related Validity and Reliability Analysis

Life satisfaction, interpersonal trust, and trust propensity were selected to measure the validity of the calibration scale, and the results showed that the total score of social trust and each factor were significantly and positively correlated with interpersonal trust, trust propensity, and life satisfaction (*r* = 0.158~0.472, *ps* < 0.05), which provides evidence for good criterion validity of STS-CA (see Table 4).

To test the reliability of the adolescent social trust scale, Sample 3 (*N*3 = 1214) was analyzed. As shown in Table 4, the overall internal consistency coefficient of the adolescent social trust scale was 0.931, which was more than 0.9, indicating that the overall scale reliability was good. The McDonald’s omega coefficients for the four dimensions ranged from 0.757 to 0.905, indicating moderate and good internal consistency of the STS-CA as a whole and the subscales.

The CR values for trust in relatives, friends, strangers and organizations were 0.743, 0.803, 0.730 and 0.894, respectively, all higher than 0.7, indicating good convergent validity (Hair et al., 2006). Meanwhile, CFA with Sample 3 revealed that almost all loadings of the 27 items (0.571~0.728) were higher than 0.6, which was satisfactory according to the criterion of 0.5 or higher proposed by Hair et al. (2006).

Moreover, this study further examined the discriminant validity among the four factors of social trust, and the results showed that the square root value of a certain factor AVE is greater than the absolute value of the correlation coefficient between this factor and other factors (*r* = 0.527~0.605), indicating that the discriminant validity of the scales was acceptable.

In addition, the study data were folded in half according to odd and even items, with 14 odd items and 13 even items. The calculated Spearman–Brown coefficient (unequal length) was 0.893, further supporting the reliability of the scale. Finally, test–retest (2-month interval) reliability analyses using Sample 4 (*N*4 = 303) showed strong correlations (*r* = 0.862 to 0.927) between the scores of each subscale at both time points. Overall, the reliability indicators of the STS-CA developed in this study met the corresponding statistical requirements, indicating that the scale has good consistency and stability.

#### 3.3.4. Measurement Equivalence Across Groups

To examine whether the four-factor structure of the STS-CA was equivalent across gender and educational stage groups, multigroup CFA were performed sequentially to evaluate configural, metric, and scalar invariance (Van den Berg et al., 2002). As shown in Table 5, the fitting indices of the configural model (CFI = 0.879, TLI = 0.867, RMSEA = 0.055, SRMR = 0.048) were lower than the recommended thresholds (CFI ≥ 0.90). In response to this limitation, partial configural invariance was examined by freeing several cross-loadings and correlated residuals suggested by modification indices. The modified model indicated significantly improved fit (CFI = 0.911, TLI = 0.907, RMSEA = 0.046, SRMR = 0.048). Subsequently, partial metric invariance and partial scalar invariance models were tested stepwise. The final partial scalar invariance model exhibited acceptable fit (CFI = 0.913, TLI = 0.908, RMSEA = 0.046, SRMR = 0.048), and the changes in fit indices across nested models were within acceptable ranges (ΔCFI = −0.004 < 0.010, ΔRMSEA = 0.001 < 0.015), supporting partial measurement invariance of the scale across gender. Similar procedures were performed for multi-group analyses across the educational stage, the model fit changes in subsequent partial invariance models were satisfactory (partial Metric invariance: |ΔCFI| = 0.013, |ΔTLI| = 0.007, |ΔRMSEA| = −0.008; partial Scalar invariance: |ΔCFI| = 0.003, |ΔTLI| = 0.004, |ΔRMSEA| = −0.006), which demonstrated that partial measurement equivalence was upheld between junior high school and senior high school students.

## 4. Discussion

The present study adhered to standardized psychological scale development procedures. Multiple methods generated initial items, with expert review guiding item selection. The scale’s psychometric properties were rigorously evaluated. The final 27-item STS-CA comprises four dimensions: trust in relatives, trust in friends, trust in strangers, and trust in organizations. Unlike existing social trust questionnaires, the STS-CA uniquely captures adolescents’ trust toward both familiar individuals (e.g., parents, relatives, friends) and broader societal elements—including generalized trust in strangers and trust in societal organizations—providing a more comprehensive assessment of adolescent social trust and offering an effective measurement tool.

Firstly, the STS-CA demonstrates good structural validity. Both EFA and CFA supported a first-order four-factor model. Fit indices met established criteria (CFI > 0.90, RMSEA < 0.08, SRMR < 0.06; Hu & Bentler, 1999), with all factor loadings exceeding 0.542, confirming a well-fitting four-factor structure. This structure aligns with established conceptualizations of social trust encompassing interpersonal trust (e.g., toward family, friends, and strangers) and institutional trust (Kwon, 2019).

Secondly, the STS-CA exhibits strong criterion-related validity. Using interpersonal trust scores, general trust propensity scores, and life satisfaction scores as criterion measures, significant positive correlations were found between social trust and interpersonal trust (*r* = 0.209), social trust and general trust propensity (*r* = 0.472), and social trust and life satisfaction—consistent with prior research (Statman, 2015; Zhang, 2020). Meanwhile, the CR values for each factor were higher than 0.7, confirming the scale’s convergent validity (Hair et al., 2006). The square root value of a certain factor’s AVE (0.606, 0.671, 0.595, 0.644) was greater than the absolute value of the correlation coefficient between this factor and other factors (*r* = 0.527~0.605), which demonstrated that the STS-CA has good discriminant validity.

Thirdly, in terms of reliability, the internal consistency coefficients and composite reliability for the STS-CA and its four sub-dimensions indicated good internal consistency (>0.700). Additionally, we retested the analysis on another sample (*N*4 = 303) after a two-month interval, and the correlation coefficients ranged from 0.862 to 0.927, suggesting that the STS-CA has excellent stability.

Finally, the STS-CA establishes critical measurement invariance. Although measurement invariance is often overlooked in previous social trust instruments, it is essential for ensuring valid group comparisons (Brown et al., 2022). The four-factor structure demonstrated configural, metric, and scalar invariance across gender and educational stage (middle school vs. high school). This indicates that the construct of social trust holds equivalent meaning and measurement properties for male and female adolescents, as well as across different educational stages.

Several limitations warrant acknowledgment. First, although forty-five adolescents from multiple regions were involved in the item generation stage, the use of convenience sampling and the relatively small sample size may limit the representativeness of the initial item pool. Moreover, recruitment was limited to selected provinces in China, which may restrict external validity and generalizability beyond these settings. Future research should recruit a larger and more diverse sample from other regions to further validate the psychometric properties of the STS-CA. Second, the exclusive focus on adolescents within a collectivist cultural context (China) restricts cross-cultural comparisons. Future studies should validate comparable measures in Western individualistic contexts to examine cultural influences on adolescent social trust development. Third, as the instrument was developed for adolescents aged 12–17, future validation with elementary school and college student populations is recommended. Finally, a longer follow-up period is advised in future studies to more thoroughly explore the stability of the constructs, even if the two-month test–retest interval offers preliminary evidence of temporal stability. Moreover, the STS-CA scale developed in this study was constructed based on the trust-based dichotomy and refined through preliminary qualitative interviews, ultimately identifying four dimensions: trust in relatives, trust in friends, trust in strangers, and trust in organizations. Although our research focuses on middle school students whose primary social environment is school, interviews revealed that adolescents typically interpret “social trust” as encompassing trust in various social entities—including relatives, friends, strangers, and organizations—while rarely explicitly mentioning “school” or “teachers” as separate trust objects. Therefore, during theoretical construction and factor analysis, we conceptualize schools as a crucial context for “organizational trust” rather than an independent dimension. Future research should explore measuring “school trust” as an independent or context-dependent construct and examine its correlations with the four dimensions of STS-CA to enrich theoretical models of adolescent social trust.

This study also makes significant theoretical and practical contributions. On the one hand, the development of the STS-CA provides a scientifically validated instrument for research on adolescents’ social trust, thereby advancing this domain. Existing social trust scales typically focus on generalized evaluations of human nature, failing to capture adolescent-specific psychological characteristics. For example, instruments like the CGSS and CFPS (Huhe, 2014; Xu et al., 2023), while measuring individual levels of social trust, neither adequately account for specific trust targets nor contextual variations. In contrast, the STS-CA offers a more comprehensive assessment of adolescents’ trust levels across diverse targets (e.g., family members, friends, strangers, and organizations) and is underpinned by a multidimensional construct. Whereas existing trust scales predominantly concentrate on individual-level or interpersonal trust dynamics (Evans & Revelle, 2008; Rempel & Holmes, 1986; Yamagishi & Yamagishi, 1994), the STS-CA broadens the measurement scope. It provides a comprehensive tool encompassing both interpersonal-level and social-level trust. On the other hand, this study has several practical implications. First, fostering social trust is one of the essential tasks in school education. Within school coexistence programs, the STS-CA could serve as an effective assessment instrument. It enables school psychologists and counselors to systematically evaluate students’ levels of social trust, and offers empirical evidence for designing more targeted intervention programs to enhance students’ trust and social adjustment. This, in turn, can assist individuals in effectively coping with social adaptation difficulties that may arise from trust deficits. Second, this study provides empirical foundations for understanding the psychological connotation and structural dimensions of social trust among Chinese adolescents. As a localized instrument with good reliability and validity, the STS-CA offers educational researchers a valuable tool to assess students’ social trust levels and to gain deeper insights into its multifaceted nature, antecedents, and developmental trajectory. Finally, given that social trust is closely associated with long-term social stability (Wray-Lake & Flanagan, 2012), the findings have important policy implications. For policymakers, social trust could be integrated as a key monitoring metric within national adolescent mental health initiatives. Its inclusion in annual mental health reports would enable the dynamic tracking of evolving trends in social trust among Chinese adolescents, which would offer an empirical foundation for evaluating and refining public policies aimed at youth development and well-being.

## 5. Conclusions

The STS-CA is a 27-item scale measuring four dimensions: trust in relatives, friends, strangers, and organizations. The scale demonstrates good reliability and validity, making it an effective assessment tool for measuring the multidimensional nature of social trust among Chinese adolescents. Crucially, the STS-CA showed measurement invariance across gender and educational stage. In sum, this study extended earlier research on the multidimensional nature of social trust by illustrating it in the Chinese adolescent population.

## Figures and Tables

**Table 1:** Item analysis results (*N* = 1018).

Item	*t*	Cohen’s *d*	*r*	Item	*t*	Cohen’s *d*	*r*	Item	*t*	Cohen’s *d*	*r*
ST1	−16.15 ***	−1.36	0.49 **	ST14	−23.41 ***	−1.97	0.63 **	ST27	−24.23 ***	−2.04	0.69 **
ST2	−19.77 ***	−1.66	0.59 **	ST15	−27.16 ***	−2.28	0.72 **	ST28	−25.29 ***	−2.13	0.68 **
ST3	−14.36 ***	−1.21	0.49 **	ST16	−18.90 ***	−1.59	0.53 **	ST29	−0.26	−0.02	0.02
ST4	−16.58 ***	−1.39	0.50 **	ST17	−24.56 ***	−2.07	0.67 **	ST30	−22.36 ***	−1.88	0.63 **
ST5	−19.54 ***	−1.64	0.60 **	ST18	−2.13 *	−0.18	0.08 **	ST31	−29.35 ***	−2.47	0.72 **
ST6	−16.42 ***	−1.38	0.53 **	ST19	−25.33 ***	−2.13	0.69 **	ST32	−25.39 ***	−2.14	0.68 **
ST7	−21.93 ***	−1.84	0.65 **	ST20	−24.50 ***	−2.06	0.68 **	ST33	−24.11 ***	−2.03	0.71 **
ST8	−21.06 ***	−1.77	0.60 **	ST21	−18.05 ***	−1.52	0.58 **	ST34	−22.72 ***	−1.91	0.67 **
ST9	−18.93 ***	−1.59	0.61 **	ST22	−20.35 ***	−1.71	0.63 **	ST35	−24.40 ***	−2.05	0.67 **
ST10	−18.48 ***	−1.55	0.59 **	ST23	−1.18	−0.10	0.07 *	ST36	−22.85 ***	−1.92	0.64 **
ST11	−21.12 ***	−1.78	0.63 **	ST24	−20.38 ***	−1.71	0.62 **	ST37	−21.82 ***	−1.83	0.67 **
ST12	−24.49 ***	−2.06	0.68 **	ST25	−25.86 ***	−2.17	0.69 **				
ST13	−20.73 ***	−1.74	0.65 **	ST26	−9.37 ***	−0.79	0.33 **				

Note. *** *p* < 0.001, ** *p* < 0.01, * *p* < 0.05; ST = social trust.

**Table 2:** Factor loading matrix (rotated) for the third EFA (*N*1 = 1018).

Item	Factor 1	Factor 2	Factor 3	Commonalities	Item	Factor 4	Commonalities
ST4	0.883			0.621	ST15	0.882	0.670
ST16	0.652			0.475	ST27	0.828	0.617
ST1	0.623			0.437	ST32	0.797	0.576
ST24	0.409			0.443	ST25	0.802	0.555
ST37	0.378			0.483	ST31	0.753	0.601
ST14		0.816		0.631	ST17	0.702	0.534
ST2		0.734		0.545	ST19	0.698	0.529
ST5		0.723		0.577	ST28	0.698	0.523
ST20		0.684		0.601	ST11	0.691	0.471
ST36		0.599		0.530	ST33	0.665	0.513
ST9			0.881	0.679	ST12	0.619	0.480
ST3			0.742	0.487	ST8	0.575	0.383
ST10			0.731	0.539			
ST22			0.701	0.566			
ST21			0.641	0.494			
Eigenvalues (after rotation)	1.243	1.617	2.399			11.091	
Explained variance (%)	4.604	5.989	8.886			41.077	
Cumulative variance contribution rate (%)	4.604	10.593	19.479			60.555	

**Table 3:** Comparison of CFA of Social Trust Scale (*N*2 = 1018).

Item	χ^2^	*df*	χ^2^/*df*	RMSEA	CFI	TLI	SRMR
Four-factor model	1319.744 ***	320	4.124	0.055	0.906	0.897	0.046
Three-factor model	1664.127 ***	321	5.184	0.064	0.874	0.862	0.054
Two-factor model	2159.303 ***	323	6.685	0.075	0.828	0.813	0.060
One-factor model	3340.230 ***	324	10.309	0.096	0.717	0.694	0.084

Note: The three-factor model is a merger of trust in relatives and trust in friends based on the four-factor model. The two-factor is the merger of trust in strangers on top of the three-factor model, and the one-factor model is the merger of the four factors. CFI = Comparative Fit Index; TLI = Tucker–Lewis index; RMSEA = Root Mean Square Error of Approximation; SRMR = standardized Root Mean Squared Residual; *** *p* < 0.001.

**Table 4:** The reliability coefficient and the correlation between social trust and the criterion variables (*N*3 = 1214).

Variables	*M* ± *SD*	Cronbach’s α	McDonald’s Omega	Test–Retest Reliability	Correlation		
					Interpersonal Trust	Trust Propensity	Life Satisfaction
Social trust	94.506 ± 14.846	0.931	0.931	0.927	0.209 **	0.472 **	0.455 **
Trust in relatives	18.870 ± 3.281	0.758	0.759	0.894	0.197 **	0.375 **	0.451 **
Trust in friends	16.961 ± 3.426	0.814	0.815	0.918	0.172 **	0.388 **	0.320 **
Trust in strangers	15.632 ± 3.069	0.743	0.757	0.862	0.175 **	0.463 **	0.286 **
Trust in the organization	43.071 ± 7.917	0.904	0.905	0.916	0.158 **	0.381 **	0.419 **

Note: ** *p* < 0.01.

**Table 5:** Results of multi-group CFA.

Model	χ^2^/*df*	RMSEA [90% CI]	CFI	TLI	SRMR	ΔCFI	ΔTLI	ΔRMSEA
Gender (*n*_male_ = 634, *n*_female_ = 580)								
Configural	1730.867 ***/634	0.055 [0.052; 0.059]	0.879	0.867	0.048	-	-	-
Partial Configural	1478.136 ***/669	0.046 [0.043; 0.050]	0.911	0.907	0.048	-	-	-
Partial Metric	1500.866 ***/670	0.047 [0.044; 0.050]	0.909	0.904	0.048	0.002	0.003	−0.001
Partial Scalar	1460.101 ***/667	0.046 [0.043; 0.049]	0.913	0.908	0.048	−0.004	−0.004	0.001
Educational stage (*n_junior high school_* = 631, *n_senior high school_* = 583)								
Configural	1735.971 ***/634	0.056 [0.052; 0.059]	0.876	0.863	0.048	-	-	-
Partial Configural	1383.651 ***/620	0.047 [0.044; 0.050]	0.914	0.903	0.045	-	-	-
Partial Metric	1539.727 ***/664	0.048 [0.045; 0.052]	0.901	0.896	0.053	0.013	0.007	−0.008
Partial Scalar	1576.054 ***/667	0.049 [0.046; 0.052]	0.898	0.892	0.059	0.003	0.004	−0.006

Note. *** *p* < 0.001. CFI = Comparative Fit Index; TLI = Tucker–Lewis index; RMSEA = Root Mean Square Error of Approximation; CI = Confidence Interval; SRMR = Standardized Root Mean Squared Residual.

## Data Availability

The datasets generated during and analyzed during the current study are available from the corresponding author on reasonable request.

## Appendix A

### Appendix A.1. The 27-Item Chinese Adolescents Social Trust Questionnaire

Construct 1: Trust in relatives

ST1: I trust that my parents usually do what they promise me.

ST4: I trust that my parents are a strong support for me when I encounter difficulties.

ST16: I consider my parents always look out for me and would never harm me.

ST24: When I go out with my relatives, I trust they will provide care and ensure my protection.

ST37: I trust that my relatives genuinely care for me and offer their support.

Construct 2: Trust in friends

ST2: I trust that my friends will keep their promises to me.

ST5: My friends will support me when I have conflicts with others.

ST14: I trust my friends to keep my secrets.

ST20: I trust my friends not to use and harm me.

ST36: When I am feeling down, I trust that my friends will usually provide comfort and companionship.

Construct 3: Trust in strangers

ST3: I trust that most strangers are trustworthy.

ST9: I trust that the majority of strangers are generally friendly.

ST10: I trust that most strangers do not intentionally harm others.

ST21: When I seek assistance from strangers, I trust that they will offer help.

ST22: I trust that most strangers typically honor their promises

Construct 4: Trust in organizations

ST8: I hold the conviction that reports disseminated by official media organizations are typically objective and factual.

ST11: I consider the systems established by the government to be beneficial for the populace.

ST12: I trust that charitable organizations effectively deliver donations to those in need.

ST15: I trust that policies enacted by the government are inherently equitable.

ST17: I maintain confidence that the government attentively addresses and manages socially concerning incidents in a timely manner.

ST19: I trust that hospitals treat all patients fairly and impartially.

ST25: I perceive the educational system as operating equitably for every student.

ST27: I hold the belief that the government prioritizes the populace’s welfare when managing public affairs.

ST28: I trust that school regulations are formulated with consideration for students’ healthy development.

ST31: I trust that schools are capable of treating and caring for the interests of each student equitably.

ST32: I consider the judiciary capable of adjudicating impartially for every individual.

ST33: I trust that commercial entities adhere to business ethics and applicable laws.
